# Effectiveness of desertliving cistanche in managing hyperlipidemic osteoporosis in ovariectomized rats through the PI3K/AKT signaling pathway

**DOI:** 10.1186/s13018-024-04890-x

**Published:** 2024-07-05

**Authors:** Jia-Yue Lin, Hao-Ming Kuang, Kuan Rong, Li Peng, Jian-Jun Kuang, Xu Yan

**Affiliations:** 1 Hunan University of Traditional Chinese Medicine, Changsha, 410208 China; 2 Hunan Academy of Chinese Medicine, No. 58 Lushan Road, Yuelu District, Changsha, Hunan Province 410006 China

**Keywords:** Bone metabolism, Hyperlipidemia, PI3K/AKt pathway, Postmenopausal osteoporosis, Vascular endothelial injury

## Abstract

**Background:**

To aim of this study is to assess the mechanism through which Desertliving Cistanche modulates the PI3K/AKT signaling pathway in the treatment of hyperlipidemic osteoporosis in ovariectomized rats.

**Methods:**

We randomly assigned specific-pathogen-free (SPF) rats into five groups (*n* = 10 per group). The normal control group received a standard diet, while the model group, atorvastatin group, diethylstilbestrol group, and treatment group were fed a high-fat diet. Four weeks later, bilateral ovariectomies were conducted, followed by drug interventions. After six weeks of treatment, relevant indicators were compared and analyzed.

**Results:**

Compared to the normal control group, rats in the model group exhibited blurred trabecular morphology, disorganized osteocytes, significantly elevated levels of bone-specific alkaline phosphatase (BALP), bone Gla-protein (BGP), total cholesterol (TC), tumor necrosis factor-α (TNF-α), and receptor activator of NF-κB ligand (RANKL). Also, the model group revealed significantly reduced levels of ultimate load, fracture load, estradiol (E2), bone mineral density (BMD), osteoprotegerin (OPG), and phosphoinositide 3-kinase (PI3K) and protein kinase B (Akt) in femoral tissue. The atorvastatin group presented with higher TC and TNF-α levels compared to the normal control group. Conversely, the treatment group demonstrated enhanced trabecular morphology, denser structure, smaller bone marrow cavities, and reduced BALP, BGP, TC, TNF-α, and RANKL levels. Furthermore, the treatment group exhibited higher levels of E2, BMD, OPG, and PI3K and Akt in bone tissue compared to the model group. The treatment group also had lower TC and TNF-α levels than the atorvastatin group. Biomechanical analysis indicated that after administration of Desertliving Cistanche, the treatment group had reduced body mass, increased ultimate and fracture load of the femur, denser bone structure, smaller bone marrow cavities, and altered periosteal arrangement compared to the model group.

**Conclusion:**

Our study revealed that Desertliving Cistanche demonstrated significant efficacy in preventing and treating postmenopausal hyperlipidemic osteoporosis in rats.

## Background

Based on statistics, the prevalence of osteoporosis among women aged 50 and older in China is 29.31%, reflecting the global aging trend [1]. Postmenopausal osteoporosis (PMOP) leads to fractures and lifelong disability and also induces high-risk chronic diseases like cardiovascular and cerebrovascular diseases, pneumonia, and diabetes, which are difficult to reverse. The treatment for PMOP reduces the quality of life, consumes medical resources, and places a significant burden on the social economy [2]. 

Corticosteroid-induced osteoporosis (CIO) is a common type of secondary osteoporosis that can result in fractures, increased morbidity, and mortality. Alendronate demonstrated superior efficacy in increasing bone mineral density (BMD) at the femoral neck and hip compared to other antiresorptive treatments [3]. A study from southern Italy indicated that the C/T-FokI single nucleotide polymorphism (SNP) of the vitamin D receptor gene may influence the response to anti-osteoporotic therapy in postmenopausal women, indicating the potential for personalized treatment based on genetic factors [4]. Moreover, addressing fragility fractures necessitates a personalized strategy, involving interdisciplinary, ortho-geriatric co-management with trauma surgeons, nurses, physiotherapists, and other healthcare professionals [5]. 

Jin Gang Wan, an ancient Chinese prescription for treating bone flaccidity, is formulated from Desertliving Cistanche. Previous studies have demonstrated several benefits of Desertliving Cistanche, including its antioxidant and anti-aging properties, promotion of bone marrow-derived mesenchymal stem cell growth in osteoporotic rat models, enhancement of cellular nutritional factors, inhibition of cell apoptosis by reducing apoptotic factors, protection of vascular endothelial function, and reduction of inflammation [6]. 

Inflammation has a crucial regulatory role in the pathogenesis of PMOP. Patients with PMOP exhibit significantly increased levels of pro-inflammatory factors in their serum [7]. Inhibiting inflammation has proven effective in treating PMOP. The PI3K/Akt signaling pathway, which is central to regulating bodily inflammation, is implicated in the mediation of the occurrence and progression of PMOP. Inhibition of the PI3K/Akt pathway and angiogenesis aggravates bone loss symptoms in PMOP.

Thus, the PI3K/Akt pathway represents a therapeutic target in PMOP. However, it remains unclear whether the pharmacological mechanisms of Desertliving Cistanche in treating PMOP involve activating PI3K/Akt signaling and inhibiting inflammation. Therefore, this study established a PMOP rat model to assess this aspect further.

## Materials and methods

### Experimental animals and grouping

Experimental animals: Fifty female specific-pathogen-free (SPF) rats, aged 10 months (procured from the Animal Experimental Center of Hunan Academy of Chinese Medicine), with a body mass of (220 ± 40) g and an animal license number: 43,602,500,000,352, were used. The rats were randomly allocated into five groups—normal control, model, diethylstilbestrol, atorvastatin, and treatment groups (*n* = 10 per group)—using a random number table. All rats were housed in SPF animal rooms with adequate ventilation and lighting, regularly changed bedding, clean cages, maintained at a constant relative humidity of approximately 50–60%, and kept at an appropriate room temperature.

The normal control group was fed a normal diet for 4 weeks, whereas the model group, atorvastatin group, diethylstilbestrol group, and treatment group were fed a high-fat diet for 4 weeks to induce the hyperlipidemic rat model. The normal diet, purchased from Beijing Keao Xieli Feed Co., Ltd., contained corn (48%), wheat flour (20%), soybean cake (15%), rice bran (12%), and fish meal (5%), and the rats were fed once daily. The high-fat diet, given twice daily, consisted of 1.25% cholesterol, 9% sucrose, 0.5% sodium cholate, 3% lard, 3% peanut oil, 5% egg yolk powder, and 78% normal diet enriched to contain 40% fat. Weekly measurements included monitoring the body weight and feed intake of rats in each group.

### Experimental drug

The primary ingredient of Jin Gang Wan is Desertliving Cistanche, supplemented with a few Rhizoma Dioscoreae, Eucommia Bark, Dodder Seed, and pig kidney, all finely powdered (120 g each) and processed by boiling in wine. These ingredients are combined and formed into pills using honey. Jin Gang Wan (manufactured by Shanxi Tianyang Pharmaceutical Co., Ltd.; specification: 3.5 g/pill; Chinese medicine approval: Z61020726; batch number: 202,004); Diethylstilbestrol Tablets (manufactured by Tianjin Lisheng Pharmaceutical Co., Ltd.; specification: 0.5 mg/tablet; Chinese medicine approval: H12020154; batch number: 219 A); Atorvastatin (manufactured by Shumaitong Pharmaceutical; specification: 10 mg/tablet; batch number: H20193043).

### Main reagents

Loading buffer 6X (batch number: CW0610), mRNA reverse transcription kit (batch number: CW2569), and miRNA reverse transcription kit (batch number: CW2141) were sourced from Kangwei Shiji Co., Ltd., Beijing, China. EDTA (batch number: MB2514) was obtained from Dalian Meilun Biotech Co., Ltd., China. The nucleic acid dye (batch number: PB11141) was purchased from Applygen Technologies Inc., Beijing, China. ELISA kits for OPG, RANKL, TC, and TNF-α specific to rats (batch numbers: mll33271, ml601105, ml003065, ml347206) were procured from Shanghai Enzyme-linked Biotechnology Co., Ltd. Primary antibodies for PI3K and AKT (batch numbers: ab1549, ab179463), OPG (batch number: ab73400), and RANKL (batch number: ab45039) were sourced from Abcam Company, UK. BCA kits and HE staining kits were purchased from Beyotime Biotech. Inc., Shanghai (article numbers: P0011, C0105, P0013K, etc.). Penicillin (batch number: L201008) was supplied by Huabei Pharmaceutical Co., Ltd.

### Main instruments

The equipment used in the study included: H1650R benchtop high-speed refrigerated centrifuge (manufactured by Hunan Xiangyi Laboratory Instrument Development Co., Ltd.); Multifunctional enzyme label analyzer (manufactured by Shenzhen Huisong Technology Development Co., Ltd.; model: MB-530); Electrothermal constant temperature incubator (manufactured by Beijing Ever Bright Medical Treatment Instrument Co., Ltd.; model: DHP-500); Shaker (manufactured by Haimen Kylin-Bell Lab Instruments Co., Ltd.; model: TS-1); Quantitative fluorescent RCP instrument (manufactured by Thermo Fisher Scientific, USA; model: PIKOREAL96); Fluorescent PCR plate (manufactured by Thermo Fisher Scientific, USA; model: SPL0960).

### Procedure for setting up the model

The model was established based on relevant literature and prior studies. Rats were positioned supine on a sterile operating table and anesthetized with intraperitoneal injection (10%, 0.3 mg/kg). Following routine disinfection, a surgical incision was made in the abdomen and securely closed with sutures. Bilateral ovariectomies were conducted on rats assigned to the model group, diethylstilbestrol group, treatment group, and atorvastatin group. Post-surgery, each rat received intramuscular injections of 50,000 units of penicillin once daily for three consecutive days to prevent infection.

### Drug treatment and sampling

Jin Gang Wan was intragastrically administered to the rats daily in the following dosages: the treatment group received 3.24 g/kg, the diethylstilbestrol group received 1.0 mg/kg, and the atorvastatin group received 1.0 mg/kg. These dosages were designed to simulate human food intake. The normal control and model groups were intragastrically administered an equal volume of normal saline daily. After continuous administration for 6 weeks, the rats were euthanized, and various indicators were assessed. Detailed records of physical activity and body mass were maintained for each group. Prior to euthanasia, 5 mL of blood was collected from the abdominal aorta of each rat, and the serum was separated and stored at -80 °C for later use.

### Detection of relevant study indicators

#### Body mass

After 6 weeks of treatment, rats in each group underwent a 12-hour fasting period without water deprivation. Their body mass was measured the following morning.

#### HE staining

Paraffin sectioning and preparation: The right tibia of the rats was fixed in 4% paraformaldehyde and then decalcified with 10% EDTA solution, with the medium changed every 3 days. Decalcification lasted for 6 weeks at room temperature, with completion indicated by the absence of resistance when pierced with an injection needle. Approximately 1 cm of the upper tibia was removed and subjected to gradient ethanol dehydration (immersed in solutions of 70%, 80%, 90%, 95%, and 100% ethanol sequentially, for 15 min each). Xylene was used for clearing the tissue sections (twice for 40 min each). The samples were embedded in paraffin with the coronal side facing outward, and the thickness of the sections was set to 5 μm. After floating the sections in a 42 °C water bath, they were mounted on slides and baked at 60 °C for 5 h for subsequent use.

Staining: Paraffin sections were deparaffinized with xylene and rehydrated based on a series of graded alcohols to water. The sections were then fixed in BOUIN fixative for 1 h and washed with running water until they turned colorless. Subsequently, the sections were stained with HE staining solution for 10 min and rinsed with distilled water until clear. A 1% phosphoric acid solution was used for 5–10 min to differentiate the staining until the trabeculae faded. The sections were then immersed in a 0.5% solid green staining solution for 5 min and dehydrated based on a graded series of ethanol (70%, 80%, 90%, 95%, and 100% ethanol, soaking in each solution for 5 min). Finally, the sections were cleared with xylene and mounted with neutral gum for microscopic assessment.

#### BMD detection

The right femoral soft tissue was removed from each bone marker, and bone mineral density (BMD) was measured with a dual-energy two-photon BMD instrument. The ultimate load and the fracture load were measured using an AG-IX biomechanical universal testing machine. The left femur of all rats was placed on a testing machine with a span of 20 mm and a constant loading speed of 10 mm/min. The probe was pressed down to the middle tibia until it fractured. The load-deformation curve was.

recorded and the data was analyzed.

#### The ELISA method

The enzyme-linked immunosorbent assay (ELISA) method was used to detect serum levels of BALP, E2, BGP, OPG, TC, RANKL, and TNF-α. The collected serum samples were analyzed using a fully automated biochemical analyzer. The process included sample addition, enzyme addition, liquid preparation, washing, and chromogenic determination of standards. The absorbance (OD value) of each well was measured sequentially at a wavelength of 450 nm, with blank wells serving as references. The measurements were conducted within 15 min after the stop solution was added.

#### Detection of PI3K and AKT protein expression in bone tissue using Western blot

One hundred milligrams of rat femur was frozen with liquid nitrogen and ground into powder. The powdered tissue was then transferred to a centrifuge tube, mixed with 300 µL RIPA cell lysate and phosphatase inhibitor, and processed in a biological sample homogenizer to ensure thorough lysis of the bone tissue. The mixture was lysed for 10 min and centrifuged at 4℃ at 12,000 r/min for 15 min (centrifugation radius: 8 cm). The supernatant was collected to obtain the total protein, and the protein concentration was measured and quantified. A protein of 40 µg was subjected to SDS gel electrophoresis for separation and then transferred to a PVDF membrane. The membrane was blocked with 5% skimmed milk powder and incubated with the primary antibody overnight at 4℃. Following this, the membrane was incubated with the secondary antibody at room temperature at 5℃ for 120 min.

For ECL chromogenic exposure, the membrane was incubated with ECL chemiluminescence solution for 1 min, the excess liquid was absorbed with filter paper, and images were captured using an imaging system instrument. Greyscale analysis was conducted using appropriate software with three replicates for each group. The reference protein used for normalization was β-actin.

### Statistical analysis

SPSS 21.0 statistical software was used for data analysis. Power calculation was conducted to determine the required sample size, ensuring a power of 0.80 with an alpha level of 0.05 for detecting a medium effect size across four groups using ANOVA. The data, which were measurement data, were expressed as mean ± standard deviation (x̄ ± s). One-way ANOVA was used to compare multiple groups, and t-tests were used for pairwise comparisons. When variance heterogeneity was present and the normal distribution assumption was not met, Dunnett’s t-test was used. Differences were considered statistically significant when *P* < 0.05.

## Results

### Body weight comparison of rats in each group

As revealed in Table 1, the model group exhibited a significantly higher body mass compared to the normal control group (*P* < 0.05). In contrast, the body mass of the diethylstilbestrol group, the treatment group, and the atorvastatin group was significantly lower than that of the model group (*P* < 0.05). Furthermore, the body mass in the treatment group was lower than that in the diethylstilbestrol group (*P* < 0.05).

**Table 1:** Comparison of body mass of rats in each group ($\overline x$*s*)

Group	*n*	Body Mass/g
Normal control group	10	175.43 ± 13.11
Model group	10	253.09 ± 27.63^*^
Diethylstilbestrol group	10	247.45 ± 27.52^*#^
Treatment group	10	234.63 ± 24.12^*#&^
Atorvastatin group	10	220.77 ± 22.13^*#&※^

Note: *P < 0.05 when compared to the normal control group; ^#^*P* < 0.05 when compared to the model group; ^&^*P* < 0.05 when compared to the diethylstilbestrol group; ^※^*P* < 0.05 when compared to the treatment group

### Comparison of total cholesterol levels in rats in each group

As revealed in Table 2, the model group’s serum TC levels were significantly higher compared to the normal control group (*P* < 0.05). All administered groups exhibited significantly lower serum TC levels than the model group (*P* < 0.05). Also, the treatment group and the atorvastatin group had significantly lower serum TC levels compared to the other groups (*P* < 0.05).

**Table 2:** Comparison of serum TC levels between groups of rats (µmol/L, $\overline x$*s*)

Group	*n*	TC
Normal control group	10	197.53 ± 34.72
Model group	10	568.76 ± 55.51*
Treatment group	10	294.20 ± 21.78*^#^
Atorvastatin group	10	291.81 ± 27.10*^#&^

Note: *P < 0.05 when compared to the normal control group; ^#^*P* < 0.05 when compared to the model group

### HE staining results

**Fig. 1:**
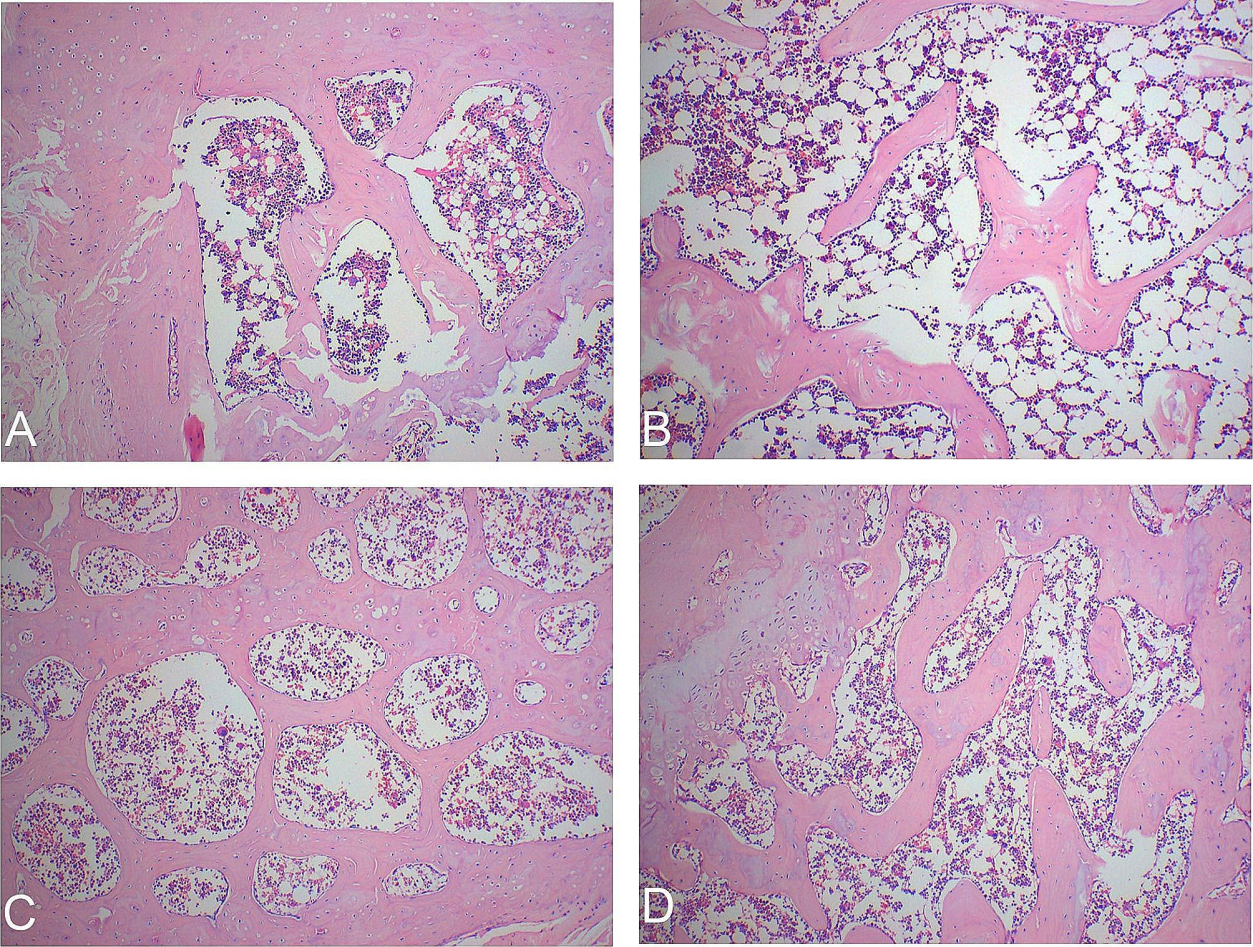
HE staining for bone histomorphology in all groups of rats. Note: **A**: Normal control group; **B**: Model group; **C**: Diethylstilbestrol group; **D**: Treatment group

As revealed in Fig. 1, the normal control group displayed intact trabecular morphology with no obvious fractures, a normal bone marrow cavity, and evenly arranged osteocytes. In contrast, the model group exhibited blurred trabecular morphology, widespread fractures, a widened bone marrow cavity, and disorganized osteocytes. The diethylstilbestrol group and the treatment group revealed enhanced trabecular morphology, a more compact structure, smaller bone marrow cavity, and a periosteum arrangement that closely resembled that of the normal control group.

### Comparison of BMD levels in rats in each group

As revealed in Table 3, the model group exhibited significantly lower BMD levels compared to the normal control group (*P* < 0.05). Both the treatment group and the diethylstilbestrol group had higher BMD levels than the model group (*P* < 0.05). Also, the treatment group demonstrated higher BMD levels than the diethylstilbestrol group (*P* < 0.05).

**Table 3:** Changes in BMD levels in various groups of rats ($\overline x$*s*)

Group	*n*	BMD/(g/cm^3^)
Normal control group	10	0.143 ± 0.005
Model group	10	0.115 ± 0.004^*^
Diethylstilbestrol group	10	0.149 ± 0.006^*#^
Treatment group	10	0.153 ± 0.004^*#&^

Note: *P < 0.05 when compared to the normal control group; ^#^*P* < 0.05 when compared to the model group; ^&^*P* < 0.05 when compared to the treatment group

### Comparison of bone biomechanics of rats in each group

As revealed in Table 4, the model group exhibited lower ultimate load and fracture load compared to the normal control group (*P* < 0.05). Both the treatment group and the diethylstilbestrol group demonstrated higher ultimate load and fracture load than the model group (*P* < 0.05). However, the treatment group had lower ultimate load and fracture load compared to the diethylstilbestrol group (*P* < 0.05).

**Table 4:** Comparison of bone biomechanical indices between groups of rats ($\overline x$*s*)

Group	*n*	Ultimate load/*N*	Fracture load/*N*
Normal control group	10	145.79 ± 0.32	142.21 ± 0.09
Model group	10	87.13 ± 0.16^*^	85.43 ± 0.12^*^
Diethylstilbestrol group	10	127.96 ± 0.27^*#^	123.17 ± 0.27^*#^
Treatment group	10	121.57 ± 2.11^*#&^	116.62 ± 1.59^*#&^

Note: ^*^P < 0.05 when compared to the normal control group; ^#^*P* < 0.05 when compared to the model group; ^&^*P* < 0.05, when compared to the diethylstilbestrol group

### Comparison of BALP, E2, and BGP levels in rats in each group

The levels of BALP, E2, and BGP in each group are presented in Table 5. The model group exhibited higher levels of BALP and BGP and lower levels of E2 compared to the normal control group (*P* < 0.05). When compared with the model group, the treatment group and the diethylstilbestrol group revealed lower levels of BALP and BGP and higher levels of E2 (*P* < 0.05). Also, the treatment group had lower BALP and BGP levels and higher E2 levels than the diethylstilbestrol group (*P* < 0.05).

**Table 5:** Comparison of BALP, E2 and BGP levels in various groups of rats ($\overline x$*s*)

Group	*n*	BALP(U/L)	E2(ng/L)	BGP(ng/mL)
Normal control group	10	95.75 ± 10.47	40.11 ± 3.35	2.46 ± 0.77
Model group	10	163.22 ± 11.11*	11.77 ± 2.15*	5.83 ± 0.53*
Diethylstilbestrol group	10	106.34 ± 15.17^#^	24.64 ± 3.31^#^	2.71 ± 1.13*^#^
Treatment group	10	97.25 ± 16.08^#&^	33.45 ± 2.02^#&^	2.50 ± 1.08*^#&^

Note: ^*^P < 0.05 when compared to the normal control group; ^#^*P* < 0.05 when compared to the model group; ^&^*P* < 0.05 when compared to the diethylstilbestrol group

### Comparison of relative serum levels of OPG, TNF-α, and RANKL in each group

The serum levels of OPG, TNF-α, and RANKL in each group are revealed in Table 6. The model group exhibited significantly higher levels of TNF-α and RANKL and lower levels of OPG compared to the normal control group (*P* < 0.05). Both the diethylstilbestrol group and the treatment group had lower levels of TNF-α and RANKL and higher levels of OPG compared to the model group (*P* < 0.05). Also, the treatment group displayed lower levels of TNF-α and RANKL and higher levels of OPG compared to the diethylstilbestrol group (*P* < 0.05). The atorvastatin group revealed lower levels of TNF-α and RANKL and higher levels of OPG than the model group (*P* < 0.05).

**Table 6:** Comparison of serum OPG, TNF-α and RANKL levels in various groups of rats ($\overline x$*s*)

Group	*n*	OPG(ng/L)	TNF-α(ng/L)	RANKL(ng/L)
Normal control group	10	2732.46 ± 104.50	108.06 ± 8.94	10.27 ± 0.86
Model group	10	1356.95 ± 68.94*	253.19 ± 3.18*	20.35 ± 0.93*
Diethylstilbestrol group	10	1907.04 ± 137.42*^#^	186.12 ± 6.52*^#^	15.09 ± 0.38*^#^
Treatment group	10	2422.91 ± 147.26*^#&^	153.47 ± 8.99*^#&^	12.84 ± 1.78*^#&^
Atorvastatin group	10	1845.30 ± 70.82*^#&※^	153.41 ± 9.67*^#&※^	16.21 ± 1.01*^#&※^

Note: *P < 0.05 when compared to the normal control group; ^#^*P* < 0.05 when compared to the model group; ^&^*P* < 0.05 when compared to the diethylstilbestrol group; ^※^*P* < 0.05 when compared to the treatment group

### Comparison of relative expression levels of signaling pathway PI3K/Akt mRNA in each group

**Fig. 2:**
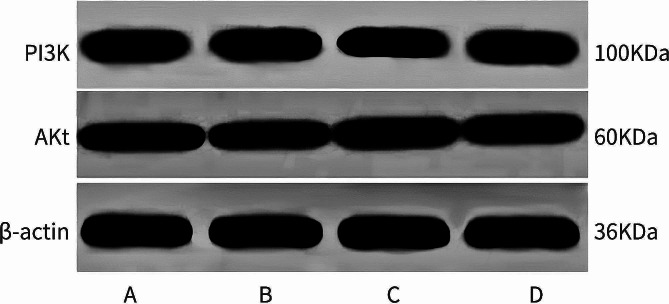
Immunoblotting of PI3K/Akt pathway proteins in bone tissue of rats in all groups Blotting results. Note: **A**: Normal control group; **B**: Model group; **C**: Diethylstilbestrol group; **D**: Treatment group

The relative expression levels of PI3K and Akt mRNA in each group are revealed in Table 7; Fig. 2. The model group exhibited significantly lower expression levels of PI3K and Akt mRNA compared to the normal control group (*P* < 0.05). The diethylstilbestrol group demonstrated significantly higher expression levels of PI3K and Akt mRNA than the model group (*P* < 0.05). Similarly, the treatment group revealed higher expression levels of PI3K and Akt mRNA compared to the model group (*P* < 0.05).

**Table 7:** The relative expression of PI3K/ akt mRNApathway protein in bone tissue of rats in each group ($\overline x$*s*)

Group	*n*	PI3K	Akt
Normal control group	10	1.20 ± 0.01	1.20 ± 0.01
Model group	10	0.24 ± 0.02*	0.43 ± 0.03*
Diethylstilbestrol group	10	0.92 ± 0.08*^#^	0.87 ± 0.09*^#^
Treatment group	10	0.96 ± 0.08*^#&^	0.89 ± 0.09*^#&^

Note: *P < 0.05 when compared to the normal control group; ^#^*P* < 0.05 when compared to the model group; ^&^*P* < 0.05 when compared to the diethylstilbestrol group

## Discussion

Based on statistics, the prevalence of osteoporosis among women aged 50 and older in China is 29.31%, reflecting a broader global trend of aging. PMOP contributes to fractures and lifelong disabilities. Also, it predisposes patients to cardiovascular and cerebrovascular diseases, pneumonia, diabetes, and other high-risk chronic diseases that are challenging to manage. These health issues not only reduce the quality of life but also deplete medical resources, imposing a substantial economic burden on society [2]. Therefore, it is crucial to develop effective strategies for the prevention and treatment of PMOP.

Pharmacologic therapies for postmenopausal osteoporosis encompass a range of treatments like bisphosphonates, SERMs, calcitonin, PTH analogues, RANK ligand inhibitors, and hormone replacement therapy. The objective of these therapies is to enhance bone density and reduce the risk of fractures [5, 8]. A recent meta-analysis of 64 randomized controlled trials involving 82,732 patients highlighted the consistent superior effects of Denosumab on bone mineral density in postmenopausal osteoporosis compared to other anti-osteoporosis medications including pamidronate, zoledronate, alendronate, and ibandronate [9]. Furthermore, Denosumab was revealed to be particularly effective in reducing non-vertebral fractures. Romosozumab emerged as highly effective in preventing vertebral fractures, while ibandronate demonstrated superior efficacy in preventing hip fractures [8]. Previous studies have assessed the predictive role of biochemical markers of bone turnover in the outcomes of postmenopausal osteoporosis therapy. These studies revealed associations between elevated levels of bone alkaline phosphatase (bALP), urinary cross-linked N-telopeptides of type I collagen (NTx), serum cross-linked C-telopeptides of type I collagen (CTx), and procollagen type I N propeptide (PINP) with increased rates of fractures and adverse events [10, 11]. 

Desertliving Cistanche exhibits antioxidant properties and anti-aging effects [6]. It targets several signaling pathways such as MAPK, VEGF, Wnt, and TNF [12–14]. In osteoporotic rat models, it promotes the growth of bone marrow-derived mesenchymal stem cells, enhances cellular nutrition, and inhibits apoptosis by reducing apoptotic factors. Also, it effectively lowers blood lipids, reduces inflammatory responses, and protects vascular endothelial function. When compared to conventional statin therapies (like atorvastatin calcium and simvastatin tablets) for hyperlipidemia, Desertliving Cistanche offers advantages in avoiding statin-related myopathy, including skeletal muscle pain, fatigue, neck pain, and joint swelling. It also reduces the risk of rhabdomyolysis and abnormal liver enzyme levels. Primarily, it is suitable for a broader range of patients, including those with liver disease who may have contraindications to statin drugs. Desertliving Cistanche is well tolerated in patients with liver disease who cannot take statins.

Through the experiments conducted in this study, notable changes were observed in the model group, including reduced trabecular density, increased trabecular spacing, enlarged bone marrow cavity, significantly elevated levels of TC and TNF-α, and significantly decreased ultimate load and fracture load of the femur. The rat models successfully replicated conditions of ovariectomy and hyperlipidemia, leading to pronounced release of the inflammatory cytokine TNF-α and substantial impairment of bone biomechanics [7]. 

The imbalance between osteoblasts and osteoclasts is a primary contributor to the disruption of bone metabolism in OP [15, 16]. The phosphatidylinositol 3-kinase (PI3K)/threonine kinase (Akt), also known as protein kinase B, and its associated signaling pathway (PI3K/Akt) play significant roles in the pathogenesis of osteoporosis [17, 18]. Xi et al. demonstrated that the PI3K/Akt pathway promotes osteoblast proliferation, differentiation, and bone formation induction for both in vivo and in vitro models of osteoporosis [19]. In this study, a decrease in PI3K and Akt mRNA expression was observed in the model group compared to the control group. Conversely, treatment with Desertliving Cistanche led to an increase in PI3K and Akt mRNA expression compared to the model group. These findings indicate that Desertliving Cistanche may prevent and treat high-fat PMOP through activation of the PI3K/Akt signaling pathway.

The OPG/RANKL/RANK (osteoprotegerin/nuclear factor-κB receptor activator ligand/receptor activator of NF-κB) system serves as the principal regulatory mechanism governing the differentiation, activation, and survival of osteoclasts [20]. This system plays a key role not only in bone metabolism but also in vascular health and the progression of atherosclerosis [21]. Specifically, the OPG/RANKL/RANK system facilitates osteoclast differentiation and enhances bone resorption activity [22]. RANK, a type II transmembrane protein within the TNF family, serves as the sole receptor responsible for regulating osteoclast development, with RANKL being the primary factor that induces the maturation of osteoclasts. Following menopause, the decline in estrogen levels in women results in reduced TNF antagonism and diminished capacity of TNF-α to inhibit osteoclast differentiation [23–26]. 

Hyperlipidemia represents a significant risk factor for both vascular endothelial injury and OP [27]. Increased cholesterol levels contribute to the overexpression of TNF-α, which leads to recurrent damage to the vascular wall. This continuous vascular damage can further lead to vascular calcification, characterized by the ongoing transport of calcium ions from the bone to the vascular wall. The molecular and biological features of vascular calcification cells closely mirror those of bone cells, exhibiting similar signaling pathways, transcription factors, and extracellular matrix interactions.

Vascular endothelial injury serves as the primary trigger for both vascular calcification and osteoporosis [27]. Arterial calcification and osteoporosis often manifest concurrently and exhibit a close correlation as indicated by conducted studies. Serum TC levels are indicative of lipid metabolism status in the body and represent a significant risk factor for cardiovascular and cerebrovascular diseases. In our study, compared to the control group, the model group exhibited elevated TC levels, which were significantly enhanced after treatment with Desertliving Cistanche.

This indicates that Desertliving Cistanche effectively improves lipid metabolism in ovariectomized rats with hyperlipidemic osteoporosis, thereby reducing the cardiovascular disease risk associated with high-fat conditions. Furthermore, our experimental results indicated that both the treatment group and the atorvastatin group exhibited notable reductions in total cholesterol levels compared to other groups. Histological examination using HE staining revealed that the diethylstilbestrol group and the treatment group exhibited improved trabecular morphology, a more compact structure, smaller bone marrow cavities, and periosteal arrangements closely resembling those of the normal control group, in contrast to the model group.

The treatment group exhibited significantly higher BMD compared to the other groups. Biomechanical comparisons indicated that the treatment group had lower ultimate load and fracture load than the other groups. Proteomic analysis revealed that PI3K and AKT expression in the femoral tissue of the model group was significantly reduced, accompanied by exacerbated pathological damage to the femoral tissue, indicating the involvement of the PI3K/AKT pathway in bone loss in rats with PMOP through its association with inflammation and metabolic disorders [28, 29]. In contrast, the treatment group demonstrated significantly increased levels of PI3K and AKT in femoral tissue, along with enhanced trabecular morphology compared to the model group. These findings indicate that Desertliving Cistanche may suppress the expression of inflammatory factors by activating the PI3K/AKT pathway, thereby ameliorating bone metabolism disorders in rats with PMOP.

In this study, compared to the normal control group, the model rats exhibited increased serum levels of TC, BALP, BGP, TNF-α, and RANKL. Conversely, they revealed lower levels of E2, BMD, OPG, PI3K, AKT, ultimate load, and fracture load. Compared to the model group, the treatment group exhibited decreased levels of TC, BALP, BGP, RANKL, and TNF-α, while demonstrating increased levels of E2, BMD, OPG, PI3K, AKT, ultimate load, and fracture load. Both the treatment group and the diethylstilbestrol group experienced significantly increased body mass. Morphologically, the model group revealed blurred trabecular morphology, widespread fractures, widened bone marrow cavities, and disorganized osteocytic arrangement. In contrast, both the treatment and diethylstilbestrol groups exhibited enhanced trabecular morphology, more compact structure, smaller bone marrow cavities, and better periosteal arrangement compared to the model group.

The interaction between RANK and RANKL strongly promotes osteoclast growth and differentiation, thereby accelerating bone resorption in vivo [30–33]. OPG, produced by osteoblasts, acts as a soluble glycoprotein that inhibits bone resorption by serving as a decoy receptor for RANKL [34]. The balance between OPG and RANKL is crucial for maintaining normal bone metabolism. Compared to the normal control group, the model group exhibited significantly lower OPG levels and a higher number of RANKL in femoral tissue, indicating a disrupted OPG/RANKL ratio which contributes to altered bone metabolism.

This study demonstrates that the PMOP model rats experienced metabolic disturbances due to an imbalance between osteoclasts and osteoblasts, leading to increased osteoclast numbers. Assessment based on the gold standard of osteoporosis assessment, BMD, revealed that the model group had significantly lower BMD compared to the normal control group.

In conclusion, this research used robust and established indicators to highlight that Desertliving Cistanche effectively enhanced biomechanical properties and BMD in PMOP hyperlipidemic model rats. This improvement was achieved by increasing estrogen levels, restoring balance in bone metabolism, and providing long-term protection to blood vessels and bones through lipid-lowering effects that inhibit inflammatory factor expression, enhance endothelial cell growth, and reduce vascular calcification. By enhancing OP prevention efficacy and minimizing vascular calcification and injury, the frequency of cardiovascular diseases may be reduced.

In this study, we discovered that Jin Gang Wan, a renowned Chinese medicine formulation containing Desertliving Cistanche for treating bone atrophy, also effectively lowers blood lipids. This finding introduces a new therapeutic option for patients with osteoporosis combined with hyperlipidemia, potentially preventing cardiovascular diseases by improving lipid metabolism and reducing associated risk factors. Desertliving Cistanche enhances OP prevention efficacy and mitigates vascular calcification and endothelial damage, opening new avenues in clinical practice for treating hyperlipidemic OP and other bone metabolism disorders. Its mechanism involves mediating the activation of the PI3K/Akt signaling pathway, inducing VEGF expression, correcting bone metabolism disorders, and downregulating inflammatory factors like TNF-α and RANKL.

Our study is limited by the lack of measurement of daily food intake among the high-fat diet groups. This omission could potentially introduce variability in diet intake as a confounding factor. Future research should incorporate precise measurements of daily food intake to ensure more controlled dietary conditions and to enhance the validity of the findings. Also, while our study demonstrated the efficacy of Desertliving Cistanche in enhancing biomechanical properties and bone mineral density in PMOP hyperlipidemic model rats, the specific molecular mechanisms underlying these effects remain to be fully assessed. Future studies should focus on investigating these mechanisms in greater detail to provide a comprehensive understanding of how Desertliving Cistanche exerts its effects.

## Data Availability

All data generated or analysed during this study are included in this article. Further enquiries can be directed to the corresponding author.

## Abbreviations

SPF: specific-pathogen-free
BALP: bone-specific alkaline phosphatase
BGP: bone Gla-protein
TC: total cholesterol
TNF-α: tumor necrosis factor-α
RANKL: receptor activator of NF-κB ligand
BMD: bone mineral density
OPG: osteoprotegerin
PI3K: phosphoinositide 3-kinase
PMOP: Postmenopausal osteoporosis
ANOVA: One-way analysis of variance
MAPK: mitogen-activated protein kinases
VEGF: vascular endothelial growth factor
