# Correction: Effectiveness of desertliving cistanche in managing hyperlipidemic osteoporosis in ovariectomized rats through the PI3K/AKT signaling pathway

**DOI:** 10.1186/s13018-024-05025-y

**Published:** 2024-08-31

**Authors:** Jia-Yue Lin, Hao-Ming Kuang, Kuan Rong, Li Peng, Jian-Jun Kuang, Xu Yan

**Affiliations:** 1 Hunan University of Traditional Chinese Medicine, Changsha, 410208 China; 2 Hunan Academy of Chinese Medicine, No. 58 Lushan Road, Yuelu District, Changsha, 410006 Hunan Province China

**Correction: Journal of Orthopaedic Surgery and Research (2024) 19: 393**

10.1186/s13018-024-04890-x

In this article the affiliation details were incorrectly given as ‘Hunan University of Traditional Chinese Medicine, Changsha 410000, China ,2 Hunan Academy of Chinese Medicine, No. 58 Lushan Road, Yuelu District, Changsha, Hunan Province 410000, China but should have been ‘‘Hunan University of Traditional Chinese Medicine, Changsha 410208, China , 2 Hunan Academy of Chinese Medicine, No. 58 Lushan Road, Yuelu District, Changsha, Hunan Province 410006, China ‘.

The original article has been corrected.

